# The gender gap in adolescent mental health: A cross-national investigation of 566,829 adolescents across 73 countries

**DOI:** 10.1016/j.ssmph.2021.100742

**Published:** 2021-01-26

**Authors:** Olympia L K Campbell, David Bann, Praveetha Patalay

**Affiliations:** University College London, UK

## Abstract

Mental ill-health is a leading cause of disease burden worldwide. While women suffer from greater levels of mental health disorders, it remains unclear whether this gender gap differs systematically across regions and/or countries, or across the different dimensions of mental health. We analysed 2018 data from 566,829 adolescents across 73 countries for 4 mental health outcomes: psychological distress, life satisfaction, eudaemonia, and hedonia. We examine average gender differences and distributions for each of these outcomes as well as country-level associations between each outcome and purported determinants at the country level: wealth (GDP per capita), inequality (Gini index), and societal indicators of gender inequality (GII, GGGI, and GSNI). We report four main results: 1) The gender gap in mental health in adolescence is largely ubiquitous cross-culturally, with girls having worse average mental health; 2) There is considerable cross-national heterogeneity in the size of the gender gap, with the direction reversed in a minority of countries; 3) Higher GDP per capita is associated with worse average mental health and a larger gender gap across all mental health outcomes; and 4) more gender equal countries have larger gender gaps across all mental health outcomes. Taken together, our findings suggest that while the gender gap appears largely ubiquitous, its size differs considerably by region, country, and dimension of mental health. Findings point to the hitherto unrealised complex nature of gender disparities in mental health and possible incongruence between expectations and reality in high gender equal countries.

## Highlights

• Girls have worse average mental health than boys across 4 measures of mental health.
• There is large heterogeneity in the size of the mental health gender gap across countries.
• The gap is most pronounced for psychological distress and life satisfaction.
• More gender equal countries have larger gender gaps in mental health.

## Introduction

Mental ill-health is a leading cause of disease burden globally (Rehm & Shield, 2019; Walker et al., 2015), and in most individuals is first experienced in childhood (Kessler et al., 2005), leading to a growing policy interest in improving adolescent mental health (Das et al., 2016). During childhood and adolescence girls tend to report substantially worse internalising mental health than boys and this gender gap increases with age during adolescence (Bolognini et al., 1996; Bradshaw et al., 2013; Cavallo et al., 2006; Kaye-Tzadok et al., 2017; Ostberg et al., 2006; Torsheim et al., 2006; West & Sweeting, 2003). This may contribute to the disproportionately higher prevalence of common mental health disorders in adult women worldwide (Albert, 2015). It is important to document and understand cross-national differences in mental health with a focus on the gender gap: doing so may help identify countries with successful cultures and/or policies which could be implemented more broadly to reduce the gender mental health gap.

Adolescence is a formative time of changing identity (Blakemore & Mills, 2014) and is commonly when emotional disorders and the gender gap in mental health emerges (Wade et al., 2002; WHO, 2020). It is a period of rapid change and exposure to new risk factors including physical changes, peer pressure, educational stress, and sexual exploration (Viner et al., 2015; WHO, 2020). It is also a time when gender becomes a more salient socialising factor and individuals develop concepts of what it means to be a man or a woman (Greene & Patton, 2020).

Despite evidence documenting a gender difference in adolescent mental health, it remains poorly understood. First, existing evidence is largely from a small number of high-income Western countries (Bradshaw & Rees, 2017; Cavallo et al., 2006; Elgar et al., 2015; Klocke et al., 2014; Looze et al., 2018; Ottova et al., 2012; Torsheim et al., 2006) and caution must be taken when generalizing their findings to non-Western, middle and low-income countries (Henrich et al., 2010). Second, studies typically use only one measure of mental health; yet it is a multidimensional concept (Steptoe, 2019). As defined by the WHO (WHO, 2018), mental health is not simply the absence of mental illness but also a state of wellbeing and lies along a continuum from ill-health to positive mental health or wellbeing. It is constituted of several weakly correlated dimensions (Huppert & Whittington, 2003) including psychological distress, life satisfaction, hedonia (positive affect) and eudaemonia (the experience of purpose and meaning in life) (Steptoe, 2019). Third, most studies examine average differences (or binary outcomes) in mental health between countries and genders, and do not explicitly examine its distribution. Understanding which part of the population distribution drives average differences may be useful to aid understanding of the nature of the gender gap and potential policy targets (Bann et al., 2020) – for instance, average gender differences may be due to a particularly high frequency of females at the severe end of the spectrum or due to differences across the entire distribution.

Cross-national comparisons can also identify factors at the country-level which are associated with mental health. Particularly, economic factors and gender equality may play a role. Poverty is considered an established risk factor for worse mental health (Elgar et al., 2015; Carol Graham & Chattopadhyay, 2013; Lund et al., 2010; WHO International Consortium in Psychiatric Epidemiology, 2000). However, income inequality is inconsistently associated with mental health, with some studies finding a correlation between higher income inequality and worse mental health (Oishi et al., 2011; Pickett & Wilkinson, 2010), whilst others find that higher income inequality correlates with better mental health (Rözer & Kraaykamp, 2013). A meta-analysis concludes that the relationship between income inequality is weak and dependent on a countries development (Ngamaba et al., 2018). It is unknown how wealth or income inequality are associated with the gender gap in mental health, and whether this differs by dimension of mental health — life satisfaction questions for example typically correlate more strongly with economic factors than affect-related questions (Graham et al., 2010).

Existing research on the association between gender equality and mental health largely yields inconsistent findings with studies demonstrating no association (Bradshaw & Rees, 2017), stronger positive associations with both male mental health (Graham & Chattopadhyay, 2013) and female mental health (Salinas-Jiménez et al., 2016), and both smaller (Salinas-Jiménez et al., 2016; Torsheim et al., 2006) and larger mental health gender gaps (Costa et al., 2001; Carol Graham & Chattopadhyay, 2013; Zuckerman et al., 2016, 2017). Tesch-Romer et al. (Tesch-Römer et al., 2008) find that the association between gender equality and the adult mental health gender gap varies with the cultural attitudes of gender equality. Where over 50% agree with the statement ‘men have more of a right to work than a woman’, the mental health gender gap is larger with greater gender equality, but where less than 50% agree, the gap is smaller in countries with greater gender equality. Zuckerman et al. (Zuckerman et al., 2017) suggest that – in a sample of largely adults across 126 countries - a quadratic relationship exists between improving societal conditions (including gender equality) and the gender gap in subjective wellbeing. They argue that as conditions improve women’s wellbeing trends downwards relative to men, but as they continue to improve, they trend upwards. Few studies, to our knowledge, have 1) explicitly examined the relationship between gender equality and the mental health gap in adolescents, 2) investigated the adolescent gender gap in a broad sample of countries including low- and middle-income countries and, 3) focused on multiple indicators of mental health.

Using a large cross-national dataset from 73 countries and economies and spanning a range of income groups, we aimed to 1) describe the gender gap across different measures of mental health (life satisfaction, psychological distress, hedonia, eudaemonia) in terms of both average and distributional differences, and 2) investigate the correlations of macro-level economic and gender equality indicators with wellbeing in boys and girls to better understand the gender mental health gap in adolescents. Consistent with previous literature we hypothesise that girls will have worse average mental health than boys across all outcomes. However, given the inconsistency of relationships between mental health and country level indicators we ask two further research questions: 1) what is the relationship between the economic indicators - GDP and income inequality - and mental health in each gender and the gender gap? 2) What is the relationship between gender equality and mental health in each gender and the gender gap?

## Methods

### Participants

We used data from the 2018 Programme for International Student Assessment (PISA) (OECD, 2018). PISA is a multi-country cross-sectional study that surveys students at age 15 on their educational attainment and characteristics of their life (OECD, 2020). PISA operates a two-stage sample design where schools are sampled with probability proportional to the size of their enrolment of 15-year olds, and students are sampled randomly with equal probability. Students are then weighted to yield a sample that is representative of the population of the country. A response rate of 80% of selected students in each school is required. Sample sizes range from 3,363 for Malta and 35,943 for Spain. Further detail on the sampling method can be found in the technical report (OECD, 2020).

In total 73 countries and participating economies were included, containing 566,829 students (49.8% girls and 50.2% boys), representing around 28 million students. Countries excluded were Singapore; Norway; New Zealand; and Israel as they did not collect the mental health measures. Subsamples that were not nationally representative were dropped, such as China. In order to investigate regional patterns, countries were grouped by region according to the World Health Organisation’s groupings (Table S1, see for example: https://www.who.int/choice/demography/euro_region/en/). The countries sampled cover a number of regions: North and South America; Europe; Eastern Mediterranean; South East Asia; and the Western Pacific Region. Unfortunately, PISA does not collect data on mental health from any African countries apart from Morocco, so we were unable to include this region in our analysis. Morocco is grouped under Eastern Mediterranean according to WHO regional groupings.

### Measures

#### Outcome variables

Life satisfaction, psychological distress, hedonia and eudaemonia (Huppert, 2014) were all measured in PISA 2018. Life satisfaction was measured by the question: “on a scale of 0–10, overall, how satisfied are you with your life as a whole these days?”, with 0 meaning not at all satisfied and 10 meaning completely satisfied. Psychological distress was assessed with responses to how often adolescents felt sad, miserable, scared, and afraid on a scale of never, rarely, sometimes, and always. Answers were scored 1–4 and summed to give an overall score ranging from 4-16. Hedonia was assessed with responses (never to always) to how often adolescents felt happy, lively, proud, joyful, and cheerful. Answers were summed to give an overall score ranging from 5-20. Eudaemonic wellbeing was measured by asking students how much they agreed on a scale of strongly disagree, disagree, agree, and strongly agree to the following statements: “my life has clear meaning or purpose”; “I have discovered a satisfactory meaning in life”; and “I have a clear sense of what gives meaning to my life”. The answers were scored and summed to give an overall score ranging from 3-12. In order to be able to compare scales each outcome was z-score standardised to have a mean of 0 and a variance of 1. Findings did not differ when examined in the original scales (data available upon request). Invariance testing showed that measures were invariant by gender, region and gender x region (Table S2). Original items can be found in the student questionnaire (OECD, 2018).

All questions were translated into the languages of participating countries by two independent linguists and then reconciled by a third to ensure consistent meaning in all countries. Further information can be found in the PISA technical report (OECD, 2020).

#### Gender

Gender was measured by students responding to the question “are you female or male?” coded 1 for female and 0 for male.

#### National level characteristics

Measures of gross domestic product (GDP) per capita and income inequality (Gini) were taken from the World Bank dataset. GDP per capita is the total economic output of a country divided by its population and is an estimate of prosperity. The Gini index is a measure of how unequal the income distribution is and ranges from 0, representing perfect equality, to 100 representing perfect inequality.

Three measures of gender equality were used in this study: the Gender Inequality Index (GII) and the newly created Gender Social Norms Index (GSNI) derived from the World Values Survey, both produced by the UNDP; and the Global Gender Gap Index (GGGI), produced by the World Economic Forum. Whilst all three use the same themes of education, health, political and economic participation they use different indicators to make these up (Table S3 for a summary of indicators). The main difference between the GII and the GGGI is that the GII is calculated in order to measure the loss in human development from gender inequality (see http://hdr.undp.org/sites/default/files/hdr2019_technical_notes.pdf). In contrast, the GGGI aims to separate gender equality from the country’s level of development by rewarding or penalizing countries based on the size of the gender gap in a particular resource regardless of the overall level of said resource (World Economic Forum, 2018). The GSNI is different from the other two as it tries to capture social norms through the proportion of people that agree or disagree with a particular statement, for example, “men make better political leaders than women do”. This allows us to test whether cultural attitudes towards gender equality are particularly important in terms of mental health outcomes.

### Analysis

We calculate country-level average differences for each standardised measure of mental health by calculating the weighted male and female mean for each country and then subtracting female average from male. Weighted means were calculated using the R package intsvy (Caro & Biecek, 2017) designed to use the PISA provided weights and to take into account the two-stage sample design. Meta-analyses using the I^2^ statistic were performed to test heterogeneity in the gender differences between regions. The I^2^ statistic quantifies the percentage of total variation across nations due to heterogeneity rather than chance (Higgins et al., 2003). To examine the distributions of mental health outcomes across the sample, weighted frequency histograms were plotted for each country for each outcome (Figs. S2–5).

To explore the association of country-level factors on mental health outcomes, we estimated Pearson’s correlations (r) and plotted the relationships between the average score for each gender by country against the 5 country-level indicators: GDP per capita, Gini, GII, GSNI, GGGI. We use multi-level linear regression in order to estimate the between country variation in different mental health outcomes and to formally statistically investigate the associations between each of our four mental health outcomes, gender and country-level factors – GDP per capita, Gini and GGGI. We use a single indicator of gender equality to avoid multicollinearity with other equality measures (Table S4). Random intercepts for countries and random slopes for gender are modelled. Using weight scaling method A proposed by Asparouhov (Asparouhov, 2006) and Carle (Carle, 2009) we adjust the final student weights by the number of individuals in each cluster divided by the sum of the sampling weights in each cluster (see (Carle, 2009), Appendix B), in order to estimate multi-level models.

As additional and sensitivity analyses, we plot quadratic country-level associations to test for non-linear associations. Secondly, to check that models are robust to the inclusion of different measures of gender equality we ran the models using the GSNI instead of the GGGI. Thirdly, we investigated if ecological findings were robust to adjustment for individual level controls - socioeconomic background, age and immigration status. Socioeconomic status was controlled for using the PISA derived economic social and cultural status (ESCS) index that is a composite measure of parental education, highest parental occupation and home possessions. Fourthly, to test if findings are robust to the removal of country outliers, we calculate cook’s distance of countries for single-level models, with countries as data points and GGGI, GDP per capita, and Gini as independent variables, and the outcome variable as the average gender gap in each mental health outcome. The 3 countries with the highest cook’s distance are removed from the final models as a robustness check.

## Results

### Do girls have worse average mental health than boys across all outcomes?

On average, girls have worse mental health across all indicators (Table 1). Life satisfaction and psychological distress have the largest mean differences between the sexes, 0.41 (0.33 s.d) and −1.1 (0.34 s.d) respectively, whereas hedonia and eudaemonia have smaller gender gaps, 0.10 (0.39 s.d) and 0.15 (0.27 s.d) respectively. The correlation matrix shows that individual-level correlations between mental health outcomes are weak-moderate - none reach 0.5 (Table 1, top half). The country-level correlations between the gender gaps (Table 1, bottom half) are all greater than 0.5 indicating that countries with large gender gaps in one outcome are likely to have large gender gaps in others.

**Table 1:**
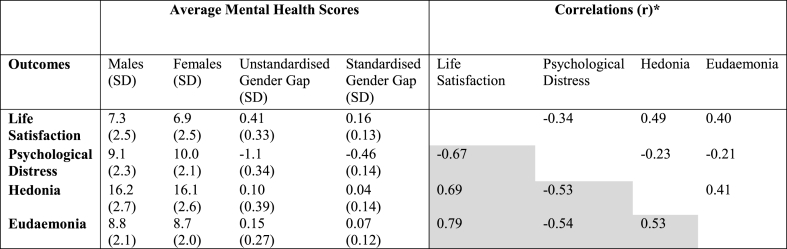
Descriptive statistics for all mental health outcomes: means; individual level correlations between mental health outcomes; and country-level correlations between the average gender gap.

Descriptive statistics for all mental health outcomes showing the mean (and standard deviation and the individual-level correlations between mental health outcomes and country-level correlations between average gender gaps. Both unstandardised and standardised mean country gender gap are shown. Note that a positive gender gap indicates worse outcomes for girls apart from for psychological distress where a negative gender gap indicates worse outcomes for girls. *the non-shaded top half of the correlation matrices contains individual-level correlations between mental health outcomes. The shaded bottom half contains country-level correlations between the average gender gaps in mental health outcomes.

**Fig. 1:**
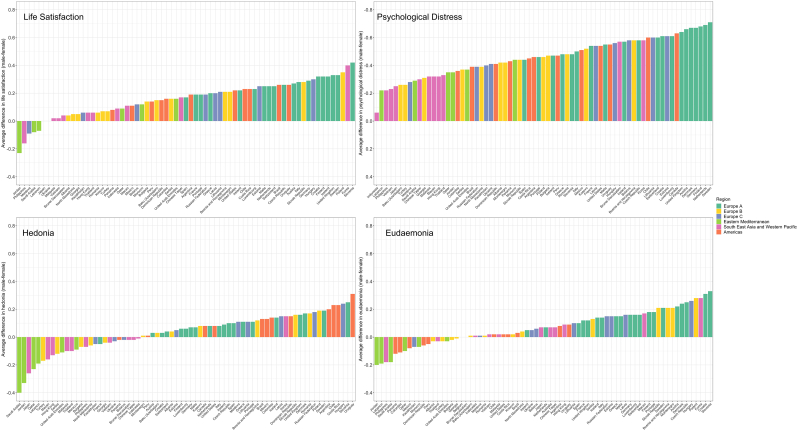
**Average standardised gender difference (male – female) in mental health across each outcome by country and coloured by region** Average gender difference in mental health outcomes (life satisfaction, psychological distress, hedonia, and eudaemonia) for each country coloured by region. Gender difference is calculated by subtracting the female from the male mean. The y-axis of the psychological distress scale is reversed to allow visual comparison with the other mental health outcomes as a more negative difference for psychological distress indicates worse outcomes for girls.

In most countries girls have worse life satisfaction, and in all countries girls report more psychological distress than boys (Fig. 1). Hedonia and eudaemonia show greater cross-cultural variation with some countries exhibiting worse average outcomes for boys, such as Jordan and Saudi Arabia (Fig. 1). Some regional patterns emerge; wealthier European nations consistently have worse average mental health for girls across all outcomes apart from hedonia; the Eastern Mediterranean countries consistently have some of the smallest gender gaps, and for hedonia and eudaemonia have better average outcomes for girls. Particular countries consistently have some of the largest gender gaps in mental health, including Sweden, Finland, Slovenia and South Korea. For each outcome there was strong evidence for heterogeneity in the gender differences - both within and between regions with I^2^ > 95% for all outcomes, p <0.001 (Fig. S1). Country distributions of mental health outcomes indicate that gender differences are driven by different parts of the wellbeing distribution; boys have higher upper values of life satisfaction (9/10 out of 10) (Fig. S2); while for psychological distress (Fig. S3) the female distribution is overall shifted to the right, indicating a higher frequency of feelings of distress in girls across the spectrum. Hedonia is also largely left skewed (Fig. S4) and the distributional gender differences are less pronounced. Eudaemonia peaks at 9 for both boys and girls in most of the countries and the gender difference looks uniform across the distribution (Fig. S4). Thus, despite different overall distributions, the mental health gender gap remains, although where the gap appears in the distribution differs by outcome.

### Country level associations

**Fig. 2:**
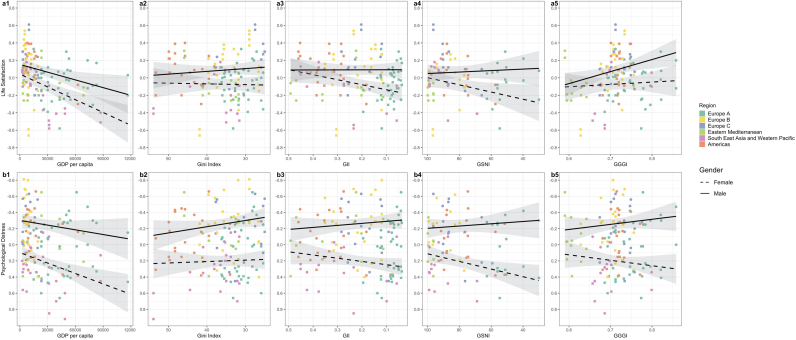
**Associations of country-level economic and gender equality indicators with average life satisfaction and psychological distress.** Fig. 2: Country-level associations of economic indicators (GDP per capita and Gini) and gender equality indicators (GII, GSNI, and GGGI) with average standardised life satisfaction (a1-5) and psychological distress (b1-5) for females and males and coloured by region. The GII, GSNI and Gini scales are reversed so that all x-axis run from less equal to more equal. The psychological distress scale is reversed so that a negative relationship indicates worse mental health across all outcomes. A larger distance between the regression lines indicates a larger gender gap. Abbreviations: Gini = income inequality, GII = gender inequality index, GSNI = gender social norms index, GGGI = global gender gap index.

The proportion of total variance attributable to differences between countries was estimated to be 5.6% for life satisfaction, hedonia and eudaemonia and 7.3% for psychological distress (using the variance partition coefficient from the baseline multi-level model (Table 2 Model A). Overall, the final model explains 37.5% of the between country variance in life satisfaction, 12.33% in psychological distress, 17.8% in hedonia, and 46.4% in eudaemonia. Fig. 2 presents the associations between the country-level indicators and each mental health outcome by gender.

**Table 2:** Regression coefficients with standard errors (SE) from multilevel models. Model A presents the baseline model to calculate the country variance partition coefficient (VPC). Model B includes only sex, Model C contains all country-level factors and Model D contains all cross-level interactions between sex female and country-level factors. The GGGI scale runs from 0-1 so we multiply it by 10 so the coefficient for GGGI represents a 0.1-point increase in the scale. GDP per capita is divided by 10,000, so that the coefficient represents the association with an increase of 1 x 10^4^ GDP per capita. Note that higher values of Gini indicate greater income inequality and that a positive coefficient for psychological distress indicates worse mental health in contrast to the other outcomes. Only the GGGI as a measure of gender equality is used due to the high correlations between the GII and GSNI and the economic variables (Table S3). *p<0.05 **p<0.01 ***p<0.001.

	Life Satisfaction Coef (SE)	Psychological Distress Coef (SE)	Hedonia Coef (SE)	Eudaemonia Coef (SE)
Model A: Baseline model				
*Country VPC*	5.6%	7.3%	5.6%	5.6%
Model B: including gender				
Female	−0.16 (0.015) ***	0.46 (0.016) ***	−0.035 (0.016)*	−0.069 (0.014)***
*Country VPC*	5.5%	8.1%	6.1%	4.3%
Model C: including country indicators				
Female	−0.16 (0.016)***	0.47 (0.017)***	−0.042 (0.017)*	−0.069 (0.015)***
GGGI * 10	0.17 (0.047)***	−0.003 (0.059)	0.15 (0.054)**	0.029 (0.04)
GDP per cap x 10^-4^	−0.032 (0.011)**	0.028 (0.013) *	−0.023 (0.012)	−0.033 (0.009) ***
Gini	−0.00003 (0.004)	0.0007 (0.004)	0.009 (0.004)*	0.003 (0.003)
*Country VPC*	3.5%	6.7%	4.6%	3.1%
Model D: cross level interactions				
Female	0.40 (0.22)	−0.18 (0.21)	1.08 (0.23) ***	0.14 (0.20)
GGGI x 10	0.17 (0.047)***	−0.069 (0.06)	0.18 (0.054)**	0.008 (0.044)
GDP per capita x^10-4^	−0.031 (0.011)**	0.013 (0.014)	−0.024 (0.012)	−0.035 (0.010)***
Gini	−0.0001 (0.004)	0.002 (0.004)	0.010 (0.004)*	0.006 (0.003)
GGGI x10 X Female	−0.081 (0.028)**	0.095 (0.027) ***	−0.16 (0.029)***	−0.065 (0.025)*
GDP per cap x 10^-4^ X Female	−0.017 (0.006)*	0.021 (0.006)***	0.006 (0.006)	−0.006 (0.006)
Gini X Female	0.002 (0.002)	−0.003 (0.002)	−0.0007 (0.002)	0.008 (0.002)***
*Country VPC*	3.5%	6.4%	4.6%	3.0%

### What is the association between the economic indicators - GDP and income inequality - and mental health outcomes in each gender?

**Fig. 3:**
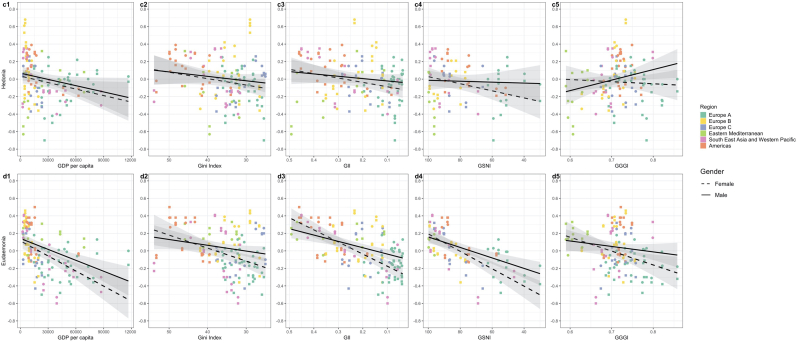
**Associations of country-level economic and gender equality indicators with average hedonia and eudaemonia.** Fig. 3: Country-level associations of economic indicators (GDP per capita and Gini) and gender equality indicators (GII, GSNI, and GGGI) with average standardised hedonia (c1-5) and eudaemonia (d1-5) for females and males and coloured by region. The GII, GSNI and Gini scales are reversed so that all x-axis run from less equal to more equal. The psychological distress scale is reversed so that a negative relationship indicates worse mental health across all outcomes. A larger distance between the regression lines indicates a larger gender gap. Abbreviations: Gini = income inequality, GII = gender inequality index, GSNI = gender social norms index, GGGI = global gender gap index.

Higher GDP per capita was associated with lower life satisfaction (β [difference in outcome per 10^4 increase in GDP per capita] 0.032 [0.01se], p<0.01), hedonia (β−0.023 [0.012se], p>0.05) and eudaemonia (β0.033 [0.009se], p<0.001) and higher psychological distress (β0.028 [0.013se], p<0.05) for both boys and girls (Figs. 2 and 3 a1-d1, Table 2 model C). For all outcomes (except hedonia) the gender gap was larger for wealthier nations mainly driven by steeper slopes for females.

Higher income inequality was associated with slightly lower life satisfaction for boys and slightly higher life satisfaction for girls and thus a slightly smaller gender gap in more unequal countries (Fig. 2: a2). Higher income inequality was associated with marginally more psychological distress for both genders (β [difference in outcome per 1 unit increase in Gini] 0.0007 [0.004se], p>0.05), but this association is slightly stronger for boys than girls and thus more equal countries have larger gender gaps (Fig. 2: b2). By contrast, lower income inequality was associated with lower hedonia (β0.009 [0.004se], p<0.05) and eudaemonia (β0.003 [0.003se], p>0.05) and slightly larger gender gaps (Fig. 3: c2 & d2). Thus, while more equal countries have larger gender gaps across all outcomes the direction of association between Gini and mental health differs by outcome.

### What is the association between gender equality and mental health in each gender?

More gender equality was associated with a larger gender gap across all mental health outcomes (Fig. 2, Fig. 3; Table 2). The processes underlying this larger gender gap differed by outcome. The larger gap in life satisfaction and psychological distress was mostly driven by positive correlations with male mental health but negative correlations with female mental health, apart from the association between GGGI and female life satisfaction which was weakly positive (β [difference in outcome per 0.1 unit increase in GGGI] 0.17 [0.047 se] in males, 0.089 in females for LS, and -0.069 [0.06se] in males and 0.026 in females for PD (Fig. 2: a5). The widening gap in hedonia and eudaemonia was mostly due to stronger negative correlations with female mental health and weaker negative correlations with male mental health, apart from the association between GGGI and male hedonia which was positive (β0.18 [0.054se] in males, 0.02 in females for hedonia, and 0.008 [0.04se] in males and -0.057 in females for hedonia) Table 2; Figs. 2 and 3). The interaction terms between GGGI and gender are large so there is fairly strong evidence that the effect for gender differs with GGGI for all mental health outcomes, apart from eudaemonia (Table 2).

### Additional and sensitivity analyses

Firstly, results were similar when analyses are controlled for age, socioeconomic status, and immigration status at the individual level (S5-8). Secondly, the interactions between gender and gender equality are robust to the use of the GSNI instead of the GGGI (Table S10). Thirdly, models and main conclusions are robust to the removal of country outliers identified by cook’s distance (Table S9). Lastly, following Zuckerman et al. (Zuckerman et al., 2017) we plot quadratic country-level associations (Figs. S6 and 7) and find that inferences drawn are largely similar to the linear associations, with richer and more gender equal countries having larger gaps. However, confidence intervals overlapped particularly for life satisfaction and psychological distress; thus, caution is required in inferring deviation from linearity.

## Discussion

Across four mental health outcomes - life satisfaction, psychological distress, hedonia, and eudaemonia - we find that girls typically had worse mental health than boys. Whilst there is considerable cross-cultural variation in the size of this average difference, it appears largely ubiquitous in this global sample - particularly for life satisfaction and psychological distress. Perhaps counterintuitively, richer European countries including the Scandinavian nations, such as Sweden and Finland, have some of the largest gender gaps in mental health. By contrast, countries with worse society gender equality scores – such as Jordan, Saudi Arabia, and Lebanon - have some of the smallest gender gaps and the direction of the gap is sometimes reversed (with boys having worse mental health). The outcomes vary in their distributions and where in the distribution the gender gap appears, indicating that mean differences are driven by different parts of the mental health distribution for the different outcomes. This highlights the importance of considering the underlying distributions of any mean differences observed. An identical mean difference may be driven by different parts of the population distribution, and this may have public health consequences. For example, we found that girls were less likely than boys to report the highest life satisfaction score, rather than having particularly higher counts in the lower part of the life satisfaction distribution. Previous research typically only focuses on mean differences – future research to understand cross-national differences in mental health may benefit from such analyses.

Higher GDP per capita was associated with a larger gender gap, albeit the magnitude of effect was small. This contrasts with other findings where a positive relationship between GDP and adolescent wellbeing has been found (Torsheim et al., 2006), and this may be due to our inclusion of a wider range of countries beyond rich Western economies. The Easterlin paradox of increasing per capita wealth not associating with increasing wellbeing is well known (Easterlin, 2003) — once basic requirements are met, material desires often increase with increasing incomes so that one is never completely satisfied (Graham et al., 2010). This however does not completely explain the negative association with mental health we found in both genders, or the larger mental health gender gap in richer countries. In line with previous literature we find an inconsistent and weak relationship between income inequality and mental health outcomes (Ngamaba et al., 2018), although it is associated with a wider gender gap in all cases. It could be the case that income inequality and GDP per capita are not particularly important amongst adolescents, and a more specific measure such as the purchasing power of adolescents might be more relevant. Or, for income inequality, the association may be dependent on a country’s level of development, with higher income inequality associating with better mental health in developing nations and worse mental health in developed nations (Ngamaba et al., 2018).

More gender equal countries had larger gender gaps across all outcomes examined, consistent with previous literature in adults (Zuckerman et al., 2017). While the gender equality measures used are not specifically designed to capture exposures directly experienced by adolescents, they reflect multiple dimensions of gender equality which influence experiences through all live stages in these countries and hence provide relevant information about the societal experiences for each gender. Whilst the nature of the associations between gender equality and adolescent mental health were inconsistent across outcomes it was striking that where the association was positive, it was particularly strong for males. This is in contrast to previous findings that show an equivalent positive relationship between gender equality and life satisfaction in boys and girls (Looze et al., 2018). Whilst previous work has shown that social norms of gender equality may be particularly important for mental health outcomes (Tesch-Römer et al., 2008) it is unclear if the multiple available gender equality indicators we used fully capture this. The newly created gender social norms index (GSNI), despite attempting to capture the distinct attitudinal aspects of gender equality, does not appear to measure gender equality in a qualitatively different way than the GII as they are highly correlated. By contrast, the GGGI captures a greater detail of gender equality by including more and more diverse indicators (Table S3), making it more granular, whilst also separating itself from a country’s level of development. For example, the GGGI includes five indicators for economic participation, such as ratio of female earned income to male, and ratio of female professional and technical workers to males, compared to the GII’s one measure of female and male labour force participation rates.

Our results present a complex picture for the relationship between gender equality and the adolescent gender mental health gap. While the feminist movement is itself old, extensive judicial and social change towards gender equality is a fairly recent development, with the UN Convention on the Elimination of all Forms of Discrimination Against Women (CEDAW) only being instituted in 1981.

Graham and Pettinato (Graham & Pettinato, 2002) coined the term ‘frustrated achievers’ to describe individuals that experience improvements in wealth but report negative perceived past mobility and lower happiness, as a result of still facing discriminatory practices and barriers to their continued ascent. In terms of women, whilst gains have been made, there remain many barriers to full equality that may explain part of our association between gender equality and worse female mental health, or only very slightly better female mental health in the case of life satisfaction. Similarly, expectations of equality may rise faster than actual experience of equality and this may result in worse mental health as women are not able to realise their goals. Another characteristic of upwardly mobile groups is that their reference categories for social comparison are usually beyond their original cohort (Easterlin, 2003). Thus, women or girls attempting to achieve the same successes as men and boys will look to them as their reference group and this may highlight the inequalities between them, producing lower life satisfaction and mental health, while in less gender equal countries reference groups might be limited to their own sex (Costa et al., 2001). Furthermore, in a number of more gender unequal countries, boys and girls might be more socially segregated at adolescence which reduce between gender comparisons.

In more gender equal countries girls and women are now faced with a double burden of balancing both increased economic and political participation as well as the traditional female responsibilities and norms. While in more gender equal countries women have entered traditionally male dominated areas of employment, men have not entered female dominated areas of employment to the same extent, nor do they do equal amounts of domestic work (England & Folbre, 2005; Garcia & Tomlinson, 2020). In countries with lower gender equality women’s roles are more fixed, whereas in more gender equal countries they are less prescribed, leading to potential conflict between roles, which may affect mental health (Hopcroft & Bradley, 2007).

Adolescence and puberty marks a particular period of changing identity (Blakemore & Mills, 2014) including developing conceptions of what it means to be a man or a woman (Greene & Patton, 2020), and while there are cross-cultural differences in experience of adolescence, identity development is common (Gibbons & Poelker, 2019). Adolescence can be particularly stressful when the norms of femininity potentially contradict with the norms of gender equality and attempting to balance the two may be additionally difficult. Previous research indicates that stress and educational pressure is particularly correlated with worse mental health in adolescent girls (Schraedley et al., 1999; Wiklund et al., 2012). Indeed, changing norms of female education and economic participation can increase educational stress and psychological distress for girls whilst they are still burdened with traditional anxieties related to maintaining a female identity and appearance (West & Sweeting, 2003) - and adolescent girls experience many more anxieties related to their appearance than boys (Smolak, 2004). Additionally, evidence suggests that individuals who violate gender stereotypes may receive backlash (Rudman et al., 2012), which may have negative consequences for mental health. Overall, adolescence marks a period of emerging new stressors which may negatively affect girl’s mental health to a greater degree than boys, and in more gender equal countries there may be more of these stressors. For example, having to balance multiple gender norms, or the stress related to the mismatch between expected and experienced gender equality and opportunities, which is potentially greater in countries perceived to have higher gender equality.

Future research should examine some of the theories we have highlighted above to better understand the individual level mechanisms. For example, to examine whether girls who attempt to satisfy multiple gender norms, such as being - femininely attractive, high achieving, and ‘one of the boys’ - have worse mental health. Additionally, examination of other country-level indicators may yield further results to help explain country-level differences in the gender gap, such as, availability and access to mental health support (Saraceno et al., 2007), levels of stigma and literacy around mental health (Corrigan & Watson, 2002), and broader factors such as estimates of environmental degradation, which may have gendered impacts (Patel et al., 2020).

### Limitations

Firstly, our study relies exclusively on cross-sectional cross-country correlations; thus, we cannot make any strong conclusions regarding the causal pathways involved. However, cross-country comparisons are necessary to elucidate risk factors that operate at the population level (Pearce, 2000), such as indicators of gender and income inequality. Secondly, whilst we cannot exclude cultural differences on likert scale responses, such as positivity biases, that may confound cross-country differences (Oishi, 2010) invariance testing of the measures indicated that the measures behaved similarly across gender and region. Thirdly, the gender gap itself may partly be a product of reporting bias – with boys being less willing to report negative mental health than girls. However, self-reports are necessary to measure mental health and wellbeing, and the extent and distributions of the gender gap being different across mental health outcomes suggests reporting biases might not be the only explanation. Fourthly, there could be systematic differences across genders in school attendance amongst the countries in our sample that could potentially bias comparison of gender gaps across countries. However, investigation of the gender ratio in secondary enrolment (obtained from the GGGI) suggests that there are not large differences in our sample. The female to male ratio in secondary enrolment ranges from 0.9 to 1.1 for our whole sample, apart from Germany (0.89), the Philippines (1.19) and Qatar (1.25). Lastly, our measure of gender was binary in nature and does not allow investigation of non-binary gender identities and mental health.

### Conclusion

Our findings demonstrate that overall girls have worse mental health than boys, but the direction and size of the gender gap and distribution varies across a range of mental health outcomes and a large sample of countries. Wealthier and more gender-equal countries, contrary to expectation, have larger mental health gender gaps. For life satisfaction and psychological distress, this was driven by negative associations in females but positive associations in males. Findings point to the complex nature of gender disparities in mental health and possible incongruence between expectations and reality in more gender equal countries.

## Ethical statement

All individual level data used in the research article is secondary data and publicly available. The mental health data is collected by the OECD's Programme for International Student Assessment (PISA). All data is anonymised by PISA. Due to the secondary nature of the data ethical clearance was not required to be sought from University College London.

The authors declare no competing interests or have any financial declarations to make.

## Credit author statement

**Olympia L K Campbell**: Formal analysis, Investigation, Writing - Original Draft, Writing - Review & Editing, Visualization

**Praveetha Patalay:** Conceptualization, Writing - Review & Editing, Formal analysis, Supervision

**David Bann:** Conceptualization, Writing - Review & Editing, Supervision

## Financial disclosure statement

There are no financial relationships between any of the authors and other organisations that may bias or influence the conclusions of this work.

## Declaration of competing interest

The authors have no affiliations or involvement with any organisations or individuals that may bias or influence the conclusions of this work. Olympia L K Campbell thanks the ESRC-BBSRC Soc-B Centre for Doctoral Training and the ERC (Grant: EvoBias 834597) for funding her PhD.
